# Evidence on nutritional therapy practice guidelines and implementation in adult critically ill patients: a scoping review protocol

**DOI:** 10.1186/s13643-019-1194-2

**Published:** 2019-11-26

**Authors:** Nomaxabiso M. Mooi, Busisiwe P. Ncama

**Affiliations:** School of Nursing and Public Health, Postgraduate Office, University of KwaZulu-Natal, Ground Floor, George Campbell Building, Howard College Campus, Durban, South Africa

**Keywords:** Critically ill patients, Nutritional therapy, Practice guidelines, Implementation

## Abstract

**Background:**

Nutritional therapy practice guidelines are designed to improve nutritional practices and thus the delivery of nutritional therapy in critically ill patients. However, they are not implemented despite the strong recommendation of nutritional therapy in the management of critical illness. The aim of this study is to map evidence on nutritional therapy guidelines and their implementation in critically ill adult patients.

**Methods:**

Two independent reviewers will conduct a search of published scholarly and gray literature on the implementation of nutritional therapy guidelines in critically ill adults using Arksey and O’Malley’s scoping review framework. The search of studies will be conducted from databases such as PubMed, Google Scholar and EBSCOhost databases, Cumulative Index for Nursing and Allied Health Literature, MEDLINE, PsychINFO, PsychARTICLES, Health Source: Consumer Edition, Health Source: Nursing/Academic Edition, PreMEDLINE, Joanna Briggs Institute, and Cochrane Databases for Systematic Reviews. We will follow a predetermined criterion to map literature and additional articles will be searched from the reference lists of included studies. The Mixed Method Appraisal Tool (MMAT) will be used for quality assessment of the included studies. Quality assessment of included studies determines the overall quality of the resultant review.

**Discussion:**

We hope to find studies on the implementation of nutritional therapy practice guidelines in adult critically ill patients and its impact on nutritional practices, patient outcomes, and health care costs. The results of this review will be disseminated through presentations in research seminars, conferences, and congresses and will also be available electronically and in print.

**Systematic review registration:**

PROSPERO CRD42017058864

## Background

Modern medicine has increased the chances of survival for many patients, which has increased the rate of critical illness [1]. Critical illness can be defined as a life-threatening multisystem process preceded by a period of increased catabolism and physiological deterioration [2]. Increased catabolism and drug-induced adverse effects, which accompany critical illness, reduce appetite, or increase nausea and vomiting making patients unable to achieve nutritional requirements by oral intake [3]. In addition, the intensive care unit (ICU) routine may also interrupt patient feeding, which warrants consideration of other methods to deliver nutritional requirements including enteral nutritional therapy (EN), parenteral nutritional therapy (PN), or a combination of both [3]. Failure to provide adequate nutritional therapy leads to malnutrition characterized by loss of lean body mass; lack of adequate physical activity and, ultimately, weakness and inability to mobilize; long periods of stay in the ICU; infectious complications; and high morbidity and mortality [4]. Therefore, means to monitor the prevalence and the indicators of critical illness-related malnutrition, emphasizing the role of nutritional therapy and the effects of nutritional therapy practice guidelines, are warranted.

### Prevalence of critical illness-related malnutrition

Thirty to fifty percent of hospitalized patients are malnourished, and the incidence is estimated to be higher in critically ill patients [1, 5]. Literature confirms that malnutrition occurs in more than 40% of ICU patients and is responsible for 50% mortality rate within 6 months following discharge from the hospital [6–8]. Consequently, malnutrition leads to high readmission rates, which impact negatively on the economic outcomes for both patients and the healthcare system [9]. The prevalence of malnutrition in critically ill patients emphasizes the need for adequate nutritional therapy and implementation of related practice guidelines [10].

### Benefits of nutritional therapy

Nutritional therapy has been proven to promote an improved nutritional status, early recovery, improved immune status, and improved quality of life following critical illness [11, 12]. It helps reduce the metabolic response to critical illness, prevent oxidative cellular injury, and favorably modulate immune response [13]. As such, societies such as the European Society of Parenteral Nutrition (ESPEN) [14] and American Society for Parenteral and Enteral Nutrition (ASPEN) [15] have published guidelines for both enteral and parenteral nutrition in ICU.

### The effects of nutritional therapy practice guidelines implementation

Nutrition therapy practice guidelines for critically ill patients are designed to help clinicians prevent malnutrition and improve patient outcomes [12, 16, 17]. However, despite the highlighted benefits, a number of barriers to effective implementation of nutritional therapy guidelines exist [18]. In many clinical settings, decisions made by healthcare providers involved in the nutrition care of critically ill patients are found not to be based on scientific evidence, leading to varied nutritional practices in many ICUs, even within a single hospital [19, 20].

Evidence-based guidelines provide recommendations based on the available evidence to address areas of ambiguity in terms of treatment options. They help clinicians make decisions regarding the feeding of critically ill patients that will contribute to the prevention of malnutrition [21]. The development of feeding protocols, which are standard operating procedures based on complex guidelines, is usually guided by these research-founded documents [22]. Guidelines are also considered as a reference to harmonize practices and enhance communication among healthcare professionals in a particular institution or setting. Adherence to such documents with institution-tailored strategies increases the efficiency of patient care among healthcare professionals with varying levels of experience and competency in nutrition therapy [22].

The poor or non-implementation of nutritional therapy practice guidelines leads to variations in nutrition therapy practices, inadequate nutrition delivery malnutrition, and resultant mortality [23]. In a case study on the practical implementation of revised nutritional therapy guidelines in the adult critically ill patient, a mention was made of some recommendations remaining unchanged due to a lack of new evidence on the topic [24]. Further, systematic reviews of the evidence are necessary to address critical outcomes for decision-making to balance risks and benefits [25]. It is hoped that the proposed review will contribute to the limited evidence on nutritional therapy practice guidelines implementation in critically ill adults and act as a baseline for future research on the topic. It also has a potential to positively influence nutritional practices and combat critical illness-related malnutrition. Therefore, this review aims to map literature on nutritional therapy guidelines in critically ill adults in order to identify gaps and provide a baseline for further research.

## Methods

This study will follow the methodological framework for scoping reviews proposed by Arksey and O’Malley and adapted by Levac et al. [26–28]. The framework has five stages that include (1) identifying the review question, (2) searching for relevant studies, (3) selecting eligible studies, (4) extracting data, and (5) collating, summarizing, and reporting of the results. However, in our review, we will identify both the research question and the eligibility criteria for the selection of studies in stage 1, as technically, the same framework that is used to identify the research question is conceived for guiding and reporting inclusion and exclusion criteria.

### Identifying the review question and eligibility criteria for the selection of studies

The population-concept-context (PCC) framework will be used to identify the main concepts of the review question and will inform the search strategy [28]. As opposed to systematic reviews, scoping reviews are helpful in answering much broader questions and the broad question this study intends to answer is, “What evidence exists on nutritional therapy guidelines and their implementation in critically ill adult patients?” [29]. Furthermore, breaking down the review question according to PCC elements allows the reviewers to check for any potentially missed inclusion and exclusion criteria in the protocol [30]. Based on the research question and the population-concept-context (PCC) framework, eligibility criteria for the selection of studies were determined to ascertain that only studies with the relevant information will be included in this review. The inclusion and exclusion criteria are shown in Table 1.

**Table 1:** Inclusion and exclusion criteria using the PCC framework

Category	Inclusion criteria	Exclusion criteria
Population	• Adult (≥ 18 years of age) critically ill patients	• Critically ill patients younger than 18 years of age • Evidence from nutritional therapy guidelines in other groups either than critically ill adults
Concept	Availability, implementation, compliance and/or adherence to: • Nutritional therapy guidelines • Nutritional support guidelines • Nutritional practice guidelines for enteral and parenteral nutrition • Artificial feeding guidelines • Standardized nutritional practices • Nutrition clinical practice guidelines • Nutritional practice recommendations • Nutritional therapy protocols, guides, procedures, and algorithms • Other reports on nutritional therapy, nutritional support, enteral nutrition, parenteral nutrition, and artificial feeding	• Studies reporting on critically ill adults on oral nutritional intake
Context	• Studies conducted in critically ill patients in the ICU, general ward, and community including home-based nutrition support • Studies conducted in any geographic setting • All types of study designs such as qualitative, quantitative, and mixed methods studies • Studies published from January 2002 to March 2019	• Studies published outside the preferred date range of this study, namely, before January 2002 and after March 2019, as guidelines of most countries were developed and published around the year 2002

### Searching for relevant studies

Published studies will be searched in databases such as PubMed, Google Scholar, Cumulative Index for Nursing and Allied Health Literature (CINAHL), MEDLINE, PsychINFO, PsychARTICLES, Health Source: Consumer Edition, Health Source: Nursing/Academic Edition, PreMEDLINE, Joanna Briggs Institute and Cochrane Databases for Systematic Reviews. Gray literature such as theses, dissertations, guidelines, policy documents, and reports and bulletins on nutritional therapy will be searched from governmental websites, online sources of organizations such as the World Health Organization (WHO), and academic databases such as ProQuest-Dissertations & Theses and Directory of Open Access Journals and Open Access Theses and Dissertations. Keywords and MeSH terms that include critically ill patients, nutritional therapy, guidelines, and implementation will be used to search for relevant studies. Keywords will be combined using Boolean search. The search results will be exported to an Endnote library that will be created for this study. An initial search was undertaken in PubMed to pilot the search strategy that has been developed for this study and the results are shown in Table 2.

**Table 2:** Pilot search strategy

Date	Keywords used	Databases searched	No. of publications retrieved	Link
January 16, 2018	Critically ill adults, nutritional therapy guidelines	PubMed	167	(("critical illness"[MeSH Terms] OR ("critical"[All Fields] AND "illness"[All Fields]) OR "critical illness"[All Fields] OR ("critically"[All Fields] AND "ill"[All Fields]) OR "critically ill"[All Fields]) AND ("adult"[MeSH Terms] OR "adult"[All Fields] OR "adults"[All Fields])) AND (("nutritional support"[MeSH Terms] OR ("nutritional"[All Fields] AND "support"[All Fields]) OR "nutritional support"[All Fields] OR ("nutrition"[All Fields] AND "therapy"[All Fields]) OR "nutrition therapy"[All Fields] OR "nutrition therapy"[MeSH Terms] OR ("nutrition"[All Fields] AND "therapy"[All Fields])) AND ("guideline"[Publication Type] OR "guidelines as topic"[MeSH Terms] OR "guidelines"[All Fields]))

### Selecting eligible studies

#### Selection process

**Fig. 1:**
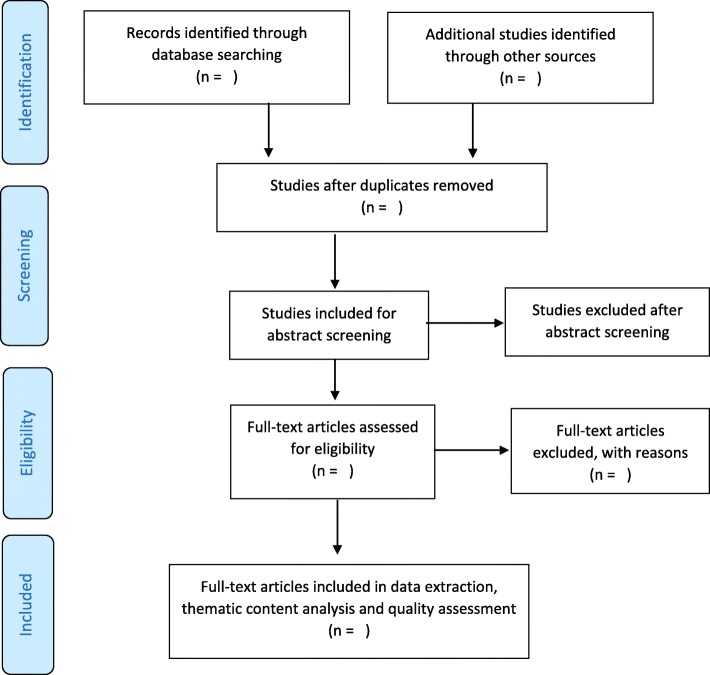
PRISMA flow diagram showing literature search and selection of studies [22, 23]

Two independent reviewers, the principal investigator and a research assistant, who have been trained as a reviewer, will engage in a rigorous three-phased process to select studies for inclusion in this review using predetermined eligibility criteria. All duplicates will be removed before screening commences. The first phase will involve title screening following the piloted search strategy. The screening of abstracts will then follow and articles agreed upon by the two reviewers will be included or excluded based on the inclusion and exclusion criteria. Any disagreements between the two reviewers will be resolved by discussion, and a third reviewer (project supervisor) will be invited for reconciliation of discrepancies where necessary. In the third stage, the full article screening will be conducted in the similar manner and reference lists of articles will also be screened for non-identified articles. Additionally, in order to maximize the search process, the authors will be consulted for full-text articles that are not available during the electronic search. An adapted PRISMA flow diagram as shown in Fig. 1 will be used to report the results of the screening process [31, 32].

### Data extraction

The reviewers will collectively develop a data charting form to determine which variables to extract to appropriately address the research question. They will use the data charting form to extract data from the first five studies independently, after which they will meet to determine if the extracted information is consistent with the overall aim of the study and to familiarize themselves with the form. The data charting form will be modified and revised as necessary during the process of extracting data from each included study. Additionally, the authors of articles will be contacted to request missing or additional data where required. Specific information that will be extracted to address the review question of this study include authors and publication date, full journal details, source or country of origin, area of care (ICU, general ward, community/home), aim of the study, study design, population, intervention, relevant results, significant results, and authors’ conclusions. Table 3 illustrates a draft of the data charting table.

**Table 3:** Draft of the data charting table

Author, publication year	Source/country of origin	Area of care (ICU, general ward, home/community)	Aim of the study	Study design	Population	Concept	Context	Relevant results	Significant findings	Authors’ conclusions

### Collating, summarizing, and reporting results

This stage will involve a descriptive summary and thematic analysis of the results of included studies, identification, and interpretation of emerging themes related to the main aim of the review and making recommendations for future research, practice, and policymaking.

1. The reviewers will numerically summarize information regarding the authors and publication dates, full journal details, source or country of origin, area of care (ICU, home, clinic), aim of the study, and study design.
2. Thematic content analysis of results on characteristics of the critically ill adults, type of guidelines and implementation, relevant results, significant findings, and authors’ conclusions will be conducted to identify emerging themes related to the purpose of the review.
3. The last step will be interpreting the emerging themes in relation to the review question and discussion of implications for future research, practice, and policymaking.

## Quality assessment

Previously, scoping reviews did not include an assessment of the methodological quality of included studies, however, that has changed; current recommendations state that the absence of quality assessment makes the results of scoping studies more difficult to interpret and limit the translation of scoping study findings into policy and practice [33, 34]. For this study, the review team will use the Mixed Method Appraisal Tool (MMAT) 2018 to determine the quality of included studies. The quality of qualitative studies will be assessed using section one of the MMAT tool. For quantitative studies, sections two, three, and four will be used for randomized controlled, non-randomized, and descriptive studies, respectively. For mixed methods studies, section one we will be used for assessing the qualitative component, while sections two, three, or four will be used for the appropriate quantitative component of a study. Section five will be used to assess quality of the mixed methods components of the included studies. Studies will be assessed for appropriateness of the study aim, study design, data collection, data analysis, relevant results, significant results, and authors’ conclusions. Further, studies will be rated as low, moderate, and high [34]. Assessment of the methodological quality of the included studies will help to determine the overall quality of the resultant review.

## Discussion

The high incidence of critical illness, advances in critical care, and improvements in patient selection have led to an increased survival of critically ill patients with high nutritional demands and resultant malnutrition [35]. Combating this double burden of malnutrition (DBM) has become a significant global health challenge in many healthcare settings [36]. DBM is characterized by the coexistence of undernutrition along with obesity or diet-related noncommunicable disease and is associated with poor outcomes and increased cost of hospitalization [20, 36]. Literature has shown that nutritional therapy, both enteral and parenteral, is the most cost-effective intervention in managing malnutrition in critical illness before ICU admission and after discharge, including the rehabilitation period [37–39]. However, nutritional practices in critically ill patients remain widely varied. This has become an indication of the need for the development and implementation of nutritional therapy practice guidelines in the form of feeding protocols as a strategy to optimize adequate delivery of nutritional therapy [40]. Societies from different countries have played a major role in the development of these guidelines for use by professionals providing nutritional therapy to pediatric and adult in/out-patients in ambulatory, home, and specialized care settings [41]. Guidelines provide basic recommendations that are supported by reviews and analyses of the current literature, to standardize and improve nutritional practices [17]. The implementation of guidelines in clinical care practice can improve patients’ nutritional status and thus contribute to achieving the Sustainable Development Goals of ending malnutrition and ensuring healthy lives and well-being for all and at all ages [42].

In a study by Barr et al. [43], 78% of patients received nutrition therapy post-guideline implementation, compared with 68% pre-implementation; however, the study does not look at the extent of availability and implementation of guidelines. Other studies emphasize monitoring of nutrition therapy, however, do not state how this can be achieved [6, 44]. Sharada and Vadivelan and Seoung-Hyun et al. believe that the implementation of nutrition guidelines can enable monitoring, standardization, and improvement of nutritional practices and thus decrease complications in critically ill patients [6, 44]. Notwithstanding the volumes of research on nutrition guidelines development, evidence on nutritional therapy guidelines in critically ill adults is lacking, hence the need for this study.

## Conclusion

The proposed scoping review will include studies that were published from 2002 to 2018 based on the trends in nutrition therapy guidelines in critically ill adults. Nutrition therapy guidelines of most countries were developed and published around the year 2002 [18, 45, 46]. Additionally, the focus has been on maternal and child nutrition, with less attention being paid to critically ill adults, which justifies the current choice of study population [47]. The results of this study may inform the development of innovative strategies to implement these guidelines, leading to reductions of the duration of critical illness and of healthcare care costs and improvement in the socioeconomic status of both patients and the state. The results of this review will be disseminated through presentation in research seminars, conferences, and congresses and will also be available electronically and in print.

## Data Availability

This is a scoping review protocol, there is no data currently, and data will be shared upon completion of the review. Only a list of references on EndNote X7 is available at the moment.

## Abbreviations

EN: Enteral nutrition
ICU: Intensive care unit
PN: Parenteral nutrition
NTG: Nutritional therapy guidelines
PCC: Population-concept-context
DBM: Double burden of malnutrition
MMAT: Mixed Methods Appraisal Tool
